# Cost-effectiveness of anti-inflammatory reliever (AIR) or maintenance and reliever therapy (MART) for mild to moderate asthma: a systematic review

**DOI:** 10.1136/bmjresp-2026-004148

**Published:** 2026-10-08

**Authors:** Nqobile Ndimande, Kevin Mortimer, Marie Stolbrink, Josue Mbonigaba, Nicola Marozva, Refiloe Masekela

**Affiliations:** 1 Department of Paediatrics and Child Health, School of Medicine, University of KwaZulu-Natal, Durban, South Africa; 2 Cambridge Africa, Department of Pathology, University of Cambridge, Cambridge, UK; 3 Respiratory Department, Aintree University Hospital, Liverpool University Hospitals NHS Foundation Trust, Liverpool, UK; 4 School of Accounting, Economics and Finance, University of KwaZulu-Natal, Durban, South Africa; 5 Department of Paediatrics and Child Health, University of Cape Town, Cape Town, South Africa; 6 Africa Health Research Institute, Mtubatuba, South Africa

**Keywords:** Asthma, Asthma Pharmacology

## Abstract

**Background:**

The Global Initiative for Asthma recommends inhaled corticosteroid (ICS)-formoterol as anti-inflammatory reliever (AIR) or as maintenance and reliever therapy (MART) for the management of all asthma severities based on evidence of superior clinical effectiveness. This systematic review aimed to collate available evidence of the cost-effectiveness of ICS-formoterol for mild to moderate asthma compared with bronchodilator and ICS, used separately or in combination, in adolescents and adults.

**Methods:**

Systematic literature searches were conducted on MEDLINE/PubMed, Scopus, Web of Science, Cochrane and the first 150 records from Google Scholar. Studies comparing the cost-effectiveness of ICS-formoterol AIR/MART to short-acting beta-2 agonist (SABA), ICS and ICS-long-acting beta-2 agonist (LABA) (other than formoterol) inhalers in people with asthma aged 12 years and older were included. Reporting was done using the Preferred Reporting Items for Systematic Reviews and Meta-Analyses (PRISMA) model.

**Results:**

Six studies evaluated as moderate to high quality involving 8901 patients were included in the analysis. Three studies were from low- and middle-income countries and three from high-income countries. ICS-formoterol AIR/MART was cost-effective when compared with SABA, ICS plus SABA and ICS-LABA plus SABA. The cost difference of ICS-formoterol ranged from an additional cost of US$56 to a cost-saving of US$9900, with small quality-adjusted life-year (QALY) gains (0.001 to 0.0077) and exacerbation reductions (up to 2.33 fewer events).

**Conclusions:**

The data suggest that ICS-formoterol AIR/MART is cost-effective for mild to moderate asthma. Due to cost-effectiveness thresholds being based on each country’s gross domestic product (GDP), more country-specific data, particularly from countries in Africa, is needed.

**PROSPERO registration number:**

CRD42024517577.

**WHAT IS ALREADY KNOWN ON THIS TOPIC**

- Although inhaled corticosteroid (ICS)-formoterol anti-inflammatory reliever (AIR)/maintenance and reliever therapy (MART) is recommended first-line treatment for asthma globally, it is not standard of care in many low- and middle-income countries due to its higher absolute cost relative to other inhalers. Evidence on the cost-effectiveness of this approach has not been previously collated in a systematic way.

**WHAT THIS STUDY ADDS**

- This systematic review provided evidence from six studies in six countries that found that ICS-formoterol AIR/MART was cost-effective when compared with short-acting beta-2 agonist (SABA) only, ICS plus SABA and ICS-long-acting beta-2 agonist plus SABA.

**HOW THIS STUDY MIGHT AFFECT RESEARCH, PRACTICE OR POLICY**

- There is low usage of ICS-formoterol AIR/MART globally despite it being recommended by international guidelines and supported by clinical effectiveness evidence. This study suggests ICS-formoterol AIR/MART may be cost-effective despite its higher unit costs. Gains are most likely due to reduced exacerbation rates and modest quality-adjusted life-year gains. The identified evidence gaps are the inclusion of more WHO regions, especially the African region, where data are lacking. Inclusion of these regions would provide representative evidence for policy makers, particularly those contemplating shifting to this approach. Our findings suggest there are cost-effectiveness as well as clinical-effectiveness arguments for ensuring all adolescents and adults with asthma globally have equitable access to ICS-formoterol AIR/MART.

## Introduction

 Asthma is a common respiratory disease globally, affecting an estimated 300 million people.^1^ Many people with asthma suffer from poor symptom control and ineffective disease management contributing to higher hospitalisation rates, medical costs and preventable deaths.^2^ Of people with asthma, approximately 45% to 70% have mild asthma with infrequent symptoms, 20% have moderate asthma with more frequent symptoms, and the remaining are categorised as having severe asthma.^2 3^ The WHO aims to reduce the prevalence and deaths due to chronic respiratory diseases by 2030 and recommends using guideline-based treatment to achieve this goal.^4^

The aims of managing asthma include achieving control of symptoms, preventing exacerbations and deaths, maintaining lung function and minimising adverse effects of treatment.^1^ This is primarily done using inhalers that can be used: (1) as required for bronchodilatation to relieve acute symptoms (short-acting beta-2 agonists (SABA)), (2) regularly (and sometimes as required) for the treatment of airway inflammation (inhaled corticosteroid (ICS)), (3) regularly in combination for both bronchodilatation and treatment of airway inflammation (ICS-long-acting beta-2 agonist (LABA)) or (4) as required for both bronchodilatation and treatment of airway inflammation in combinations of ICS-SABA and ICS-formoterol.^1^ Formoterol is a LABA that has a rapid onset of action and can therefore be used for longer duration bronchodilatation and as a reliever.^5^ Several randomised controlled trials have provided evidence of the superior clinical effectiveness and safety of ICS-formoterol, used as an anti-inflammatory reliever (AIR) or as maintenance and reliever therapy (MART), as it reduces the number of exacerbations compared with ICS, ICS plus SABA or ICS-LABA single or combination therapies.^6–10^ ICS-formoterol is now recommended by the Global Initiative for Asthma (GINA) across all asthma steps.^1^

Although ICS-formoterol AIR/MART is widely recommended, it is not the standard of care in many low- and middle-income countries (LMICs) partly due to its higher unit cost relative to other inhalers.^11^ Many patients, especially in LMICs, have limited availability and affordability of ICS-formoterol and other combination inhalers, as shown by a recent cross-sectional survey of 60 LMICs which found that the most available and affordable medication was SABA.^12^ The low availability and usage of the effective ICS-formoterol contributes to poor disease management, and to poorer outcomes, higher hospitalisation rates, increased costs and deaths due to asthma.^5 13 14^

It is more efficient for payers and policy makers to consider overall cost-effectiveness when choosing among treatment options instead of only absolute cost. Recently, there have been cost-effectiveness studies conducted that investigated the impact of using ICS-formoterol on other treatment costs and health outcomes.^15^ A systematic review which collates these cost-effectiveness data from different settings would be useful in informing decision-making and efficient healthcare resource allocation to reduce the burden and cost of asthma. This systematic literature review therefore aimed to document the global evidence on the cost-effectiveness of ICS-formoterol AIR/MART for mild to moderate asthma compared with bronchodilator and anti-inflammatory inhalers, used separately or in combination, in adolescents and adults.

## Methods

The systematic review was registered with PROSPERO (registration number CRD42024517577).

### Literature search strategy for identification of studies

Systematic literature searches were conducted on MEDLINE/PubMed, Scopus, Web of Science and Cochrane. We also assessed the first 150 records from Google Scholar. Free-text terms and Medical Subject Headings (MeSH) were used in the search strategy. The search terms related to “cost-effectiveness”, “asthma”, “adult”, “adolescent”, “ICS-formoterol” and variations of these were used. The search strategy was developed in MEDLINE and adapted for other databases (online supplemental appendix 1). Search results were exported to EndNote reference manager. Previous systematic reviews, related articles listed by search databases and reference lists of retrieved studies were used to identify additional original articles.

Two reviewers (NN and MS) independently screened the articles using a three-stage review based on eligibility criteria with initial screening by title, followed by abstract and then full-text articles. Duplicates were identified and removed. The full text of potentially eligible studies was retrieved and evaluated against the predefined population, intervention, comparator, outcome and study design criteria to identify papers for inclusion (table 1). A third reviewer (RM) was available at each stage in case of disagreements. The number of articles screened at each stage and selected for inclusion was recorded and reported using the Preferred Reporting Items for Systematic Reviews and Meta-Analyses (PRISMA) model.^16^

**Table 1:** Criteria for study eligibility

Parameter	Inclusion criteria	Exclusion criteria
Population	Adolescents aged 12 years and older and adults diagnosed with mild to moderate asthma.	Severe asthma.
Intervention	Combination ICS-formoterol, used as needed (AIR) or as both maintenance and reliever therapy (MART).	Studies with no ICS-formoterol treatment.
Comparison	Inhaled therapies used separately: short-acting beta-2 agonist (SABA), ICS or combination ICS and slow onset long-acting beta-2 agonist (LABA) (other than formoterol).	Studies with no inhaler treatments.
Outcomes	Cost-effectiveness of ICS-formoterol AIR/MART when compared with separate inhaled SABA or ICS or ICS-LABA from payer and societal perspectives.	Studies reporting only clinical effectiveness.
Time	Articles published from 1 January 2013 to 31 May 2026.	
Study design	Randomised controlled studies, non-randomised controlled studies, modelling and comparative-observational studies.	Reviews, protocols, non-comparative studies, expert opinions
Other reasons	Study type: Original research. Language: Only articles in English with full text available	Study type: Conference abstracts or unpublished material

AIR, anti-inflammatory reliever; ICS, inhaled corticosteroid; MART, maintenance and reliever therapy.

### Study selection

Articles published between 1 January 2013 and 31 May 2026 in English with full text available were included to get recent data. Studies comparing the cost-effectiveness or utility of ICS-formoterol AIR/MART to separate inhaled SABA, ICS and ICS-LABA in adolescents aged 12 years and older and adults were included. Cost-effectiveness outcomes linked cost outcomes to any clinical outcomes measured by the studies including exacerbations, asthma control test scores, change in forced expiratory volume in the 1 s and quality of life. Outcomes from all analytical perspectives reported by studies and all cost measures including direct costs, indirect costs, cost-savings and the incremental cost-effectiveness ratio (ICER), a ratio that shows the additional cost per additional health benefit of using an intervention versus an alternative, were included.^17^

### Risk of bias assessment

Eligible studies were assessed for risk of bias using the Joanna Briggs Institute (JBI) Critical Appraisal Checklist for Economic Evaluations as most studies were model based, therefore this was the best tool to use.^18 19^ The two reviewers independently assessed study quality before data were included in the analysis.

### Data extraction

Data were recorded by NN and cross-checked by MS using a standardised data extraction form and consisted of publication characteristics, study population, country, study design, intervention, comparator, perspective, sample size, time horizon, currency, type of cost-effectiveness measurement used, results and limitations.

### Data analysis and synthesis

Data on the cost-effectiveness of ICS-formoterol were extracted. A descriptive synthesis of findings from included studies in different countries, populations, study designs, perspectives and types of outcomes measured was done. Cost data were converted to 2024 US$ for comparability using the Campbell & Cochrane Economics Methods Group and the EPPI Centre Cost Converter. Treatment was considered cost-effective if its ICER was below the predefined threshold or if it saved costs while improving health outcomes when compared with another treatment.

## Results

### Study identification

We identified 2157 unique records in our literature search conducted until June 2026. We excluded 2102 records based on title and abstract screening, leaving 55 records for detailed full-text eligibility assessment. A further three records were retrieved based on reference lists of other screened records and two records from forward citation tracking using Scopus, resulting in a total of 60 full-text records. The full-text review resulted in eight studies being deemed eligible for inclusion in the review based on eligibility criteria (figure 1).

**Figure 1:**
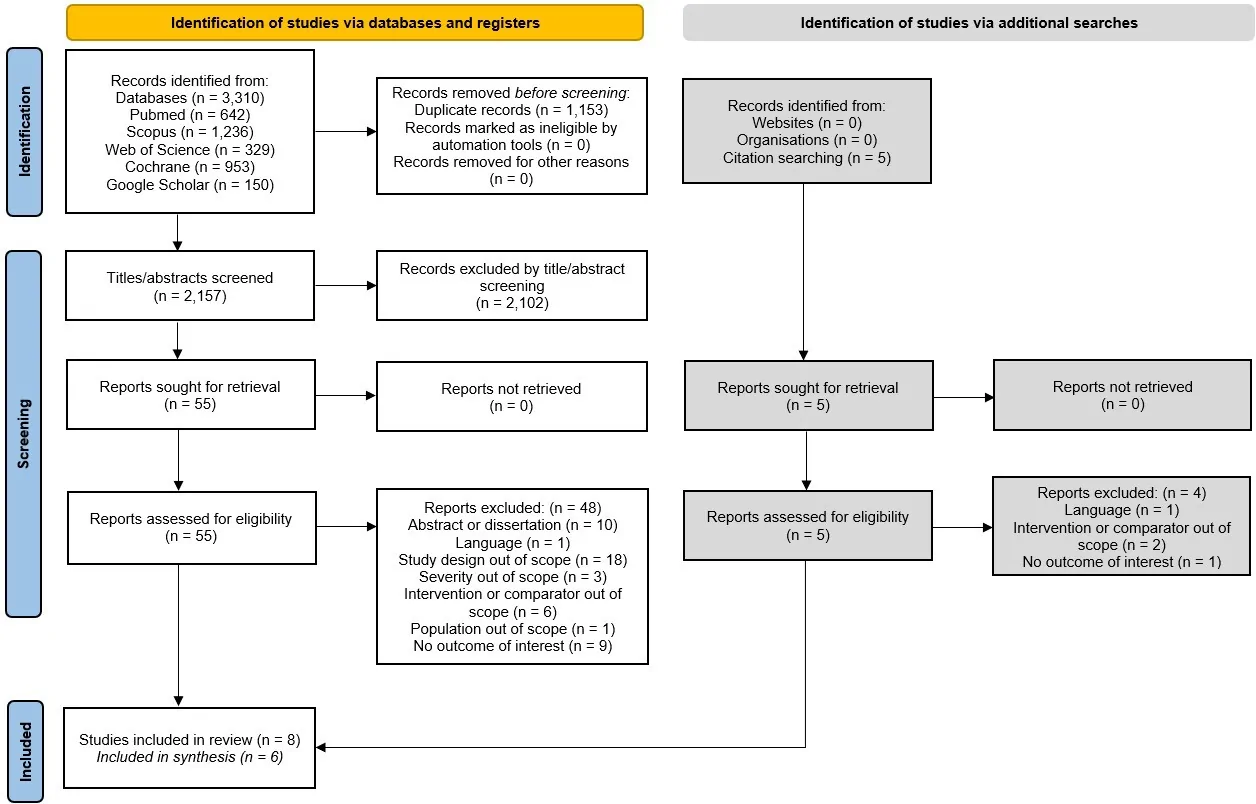
Preferred Reporting Items for Systematic Reviews and Meta-Analyses (PRISMA) flow diagram.

### Risk of bias for included studies

Based on the JBI risk evaluation, studies were classified as high quality if they scored >75%, moderate quality if they scored 60% to 75%, and low quality if they scored <60%. Two of the eight studies were assessed as low quality and were therefore excluded from the final analysis (online supplemental appendix 2).^20 21^ These studies did not satisfy the criteria regarding costs and outcomes being measured accurately and valued credibly. The studies did not provide sufficient detail on cost components and did not adequately justify the selection of data sources or comprehensively address issues of concern to end users. Six studies were assessed as high or moderate quality and were included in the final evidence synthesis (table 2). These included studies provided sufficient detail on costs and justified data sources, resulting in accurate and credible cost and outcome measurements. They also had a well-defined question and used the analysis to answer this question comprehensively to address all issues of concern to users.

**Table 2:** Characteristics of studies included in analysis

Study	Population	Comparators	Study type	Country	Perspective (primary)	Time horizon	Risk score
FitzGerald *et al*,^22^ 2020	Mild asthma aged 12 years and older	Budesonide-formoterol as needed vs Beclomethasone maintenance plus as-needed salbutamol	Markov model	UK	Payer (NHS)	Lifetime (70 years)	91%
Sadatsafavi *et al*,^23^ 2021	Mild asthma aged 12 years and older	Budesonide-formoterol as needed vs Fluticasone maintenance plus as-needed salbutamol	Markov model	Canada	Public Payer	Lifetime (50 years)	100%
Raja Gopal *et al*,^24^ 2021	Mild asthma aged 12 years and older	Budesonide-formoterol as needed vs Salbutamol as needed, OR Budesonide maintenance plus as-needed salbutamol	Decision analytical model	Malaysia	Payer (MOH)	1 year	82%
Zhou *et al*,^26^ 2024	Moderate asthma aged 12 years and older	Budesonide-formoterol reliever and maintenance vs Fluticasone-salmeterol plus salbutamol as reliever therapy	Markov model	China	Society	Lifetime	82%
Rodríguez-Martínez, *et al*,^25^ 2025	Paediatric patients aged 12 years and older with mild asthma	ICS‐formoterol as needed vs Beclomethasone and albuterol in separate inhalers as needed	Markov model	Colombia	Payer (third‐party)	12 months	73%
Leung *et al*,^27^ 2026	Mild asthma aged 18 years and older	Budesonide-formoterol as needed vs Salbutamol as needed, OR Budesonide maintenance plus as-needed salbutamol	Within-randomised controlled trial	New Zealand	Payer (health-system)	12 months	82%

ICS, inhaled corticosteroid; MOH, Ministry of Health; NHS, National Health Service.

### Studies included in the analysis

Six studies met both the eligibility and quality criteria and were included in the final analysis (table 3).^22–27^ Four involved adolescents 12 years and older and adults,^22–24 26^ one involved only adolescents over 12 years old^25^ and one involved adults aged 18 years and older.^27^ In total, 8901 patients were included in the studies, with the sample size ranging from 164 to 6735. Four studies used Markov models, one used a decision analytical model in the analysis, and one was a within-trial analysis. Five studies compared ICS-formoterol AIR with SABA and ICS plus SABA.^22–25 27^ One study compared ICS-formoterol MART with ICS-LABA plus SABA reliever therapy.^26^ Five studies used the payer perspective with only direct costs included, and one study used the society perspective including both direct and indirect costs for the primary analysis. Four studies used quality-adjusted life-year (QALY) gains as a clinical outcome, while two used exacerbations avoided for the primary analysis. Three studies used a lifetime horizon, and three used a 1-year time horizon. Three studies were conducted in LMICs, Colombia, China and Malaysia and three in high-income countries, Canada, New Zealand and the UK. Four of the studies declared funding by a pharmaceutical company.

**Table 3:** Results of synthesised studies

Study	Health outcomes	Cost outcomes (converted to 2024 USD)	ICER	Study conclusion
FitzGerald *et al*,^22^ 2020	QALY for: Budesonide-formoterol=18.88 Beclomethasone plus salbutamol=18.879 QALY gain=0.001 QALY over lifetime Secondary outcome: Exacerbations avoided=0.411 during lifetime.	Budesonide-formoterol=US$2371 Beclomethasone plus salbutamol=US$2898 Cost saving=US$527/patient over lifetime.	Budesonide-formoterol dominant, ICER not calculated.	Budesonide-formoterol can be considered cost-effective for treating mild asthma from UK healthcare payer perspective.
Sadatsafavi *et al*,^23^ 2021	QALY for: Budesonide-formoterol=25.923 Fluticasone plus salbutamol=25.921. QALY gain=0002 QALY over 50 years.	Budesonide-formoterol=US$36 504 Fluticasone plus salbutamol=is US$46 404 Cost saving=US$9900/patient over 50 years.	Budesonide-formoterol dominant, ICER not calculated.	Budesonide-formoterol is cost-effective for treating mild asthma from Canadian public payer perspective.
Raja Gopal *et al*,^24^ 2021	Severe exacerbation probability for: Budesonide-formoterol=0.0676 Salbutamol=0.1813 Budesonide plus salbutamol=0.0861.	Budesonide-formoterol=US$632 Salbutamol=US$742 Budesonide plus salbutamol=US$622 Cost saving=US$110 vs salbutamol Cost addition=US$10 vs budesonide plus salbutamol.	Budesonide-formoterol dominant vs salbutamol, ICER not calculated. US$569 per severe exacerbation averted vs budesonide plus salbutamol.	Budesonide-formoterol is dominant vs salbutamol and likely cost-effective vs budesonide plus salbutamol for treating mild asthma from Malaysian healthcare payer perspective.
Zhou *et al*,^26^ 2024	QALY for: Budesonide-formoterol=15.6643 Fluticasone-salmeterol plus salbutamol=15.6566 QALY gain=0.0077 over lifetime Secondary outcome: Exacerbations avoided=2.33.	Budesonide-formoterol=US$28 359 Fluticasone-salmeterol plus salbutamol=US$28 303 Cost addition=US$56 over lifetime.	US$7316/QALY Threshold: US$74 023/QALY.	Budesonide-formoterol is likely cost-effective for treating asthma from Chinese societal perspective.
Rodríguez-Martínez, *et al*,^25^ 2025	QALY for: Budesonide-formoterol=0.9367 Beclomethasone and albuterol=0.9352 QALY gain=0.0014.	Budesonide-formoterol=US$504 Beclomethasone and albuterol=US$779 Cost saving=US$275.	Budesonide-formoterol dominant, ICER not calculated.	Budesonide-formoterol in single inhaler is cost-effective for treating mild asthma from Colombian perspective.
Leung *et al*,^27^ 2026	Severe exacerbations rate for: Budesonide-formoterol=0.04 Salbutamol=0.11 Budesonide plus salbutamol=0.09.	Budesonide-formoterol=US$104 Salbutamol=US$98 Budesonide plus salbutamol=US$112 Cost addition=US$5 vs salbutamol Cost saving=US$8 vs budesonide plus salbutamol.	US$74 per severe exacerbation averted vs salbutamol Budesonide-formoterol dominant vs budesonide plus salbutamol, ICER not calculated. Threshold=US$148 296/QALY.	Budesonide-formoterol costs extra vs salbutamol, is cost saving vs budesonide plus salbutamol from New Zealand payer perspective and reduces exacerbations.

ICER, incremental cost-effectiveness ratio; QALY, quality-adjusted life-year.

### Review of the cost-effectiveness of ICS-formoterol AIR

ICS-formoterol used as-needed was investigated in five studies.^22–25 27^ Four had budesonide as the ICS while one did not specify the ICS molecule.

#### AIR versus SABA as required

Budesonide-formoterol as-needed was compared with salbutamol as required in two studies. Budesonide-formoterol had cost savings of US$110 when compared with salbutamol over a 1-year time horizon for participants aged 12 years and older in Malaysia. Drug costs contributed 0.2%–18% of total costs, while disease management costs from clinic visits, emergency department visits or hospitalisation due to exacerbations contributed 82%–99% of total costs for the different arms. Budesonide-formoterol also had a lower probability of severe exacerbations (0.0676 vs 0.1813), which caused total costs to be 14.9% lower than for salbutamol, making it a dominant treatment.^24^ For participants aged 18 years and older from a New Zealand health system perspective, budesonide-formoterol had an ICER of US$74 per severe exacerbation avoided when compared with salbutamol and was likely to be cost-effective. From the secondary societal perspective, budesonide-formoterol had cost savings of US$84 and was the dominant treatment.^27^ Budesonide-formoterol was therefore cost-effective for treating mild asthma when compared with salbutamol as required.

#### AIR versus ICS maintenance plus SABA as required

Budesonide-formoterol as-needed was compared with budesonide maintenance with SABA as required in four studies. Over a lifetime horizon, budesonide-formoterol was dominant with cost savings of US$527 compared with beclomethasone plus salbutamol and US$9900 compared with fluticasone plus salbutamol, with small but positive QALY gains of 0.001 and 0.002 from the UK and Canadian payer perspectives, respectively. The largest contributors to cost savings for budesonide-formoterol were drug costs (86% and 37% of total saving in the UK and Canada, respectively), due to lower doses required when using budesonide-formoterol, and adverse event related costs (63% of total saving in Canada).^22 23^ Over a 1-year time horizon, from a Malaysian payer perspective, budesonide-formoterol had an ICER of US$569 per severe exacerbation avoided when compared with maintenance budesonide plus salbutamol reliever and was likely to be cost-effective.^24^ Over the same period, from a New-Zealand health system perspective, budesonide-formoterol resulted in cost savings of US$8 in the base case and less severe exacerbations. The cost savings were 80% higher from a societal perspective.^27^ Budesonide-formoterol was therefore cost-effective when compared with budesonide plus SABA as required.

#### AIR versus SABA as required with separate ICS

ICS-formoterol in a single inhaler was compared with as-needed ICS and albuterol in separate inhalers taken at the same time from a Colombian payer perspective. Over a 1-year time horizon, ICS-formoterol in a single inhaler had cost savings of US$275 and a small gain in QALY of 0.0014 when compared with as-needed ICS and albuterol in separate inhalers in paediatric patients aged 12 years and older with mild asthma.^25^ ICS-formoterol was therefore cost-effective when compared with as-needed ICS and albuterol in separate inhalers.

### Review of the cost-effectiveness of ICS-formoterol MART

#### MART versus ICS-LABA plus SABA

Budesonide-formoterol was used as MART in one study and compared with maintenance fluticasone-salmeterol plus salbutamol reliever.^26^ Budesonide-formoterol resulted in fewer exacerbations (13.56 vs 15.89) and a QALY gain of 0.0077 at an additional cost of US$56 over a lifetime versus fluticasone-salmeterol plus salbutamol. Drug costs for budesonide-formoterol were higher than for fluticasone-salmeterol plus salbutamol; however, this was largely offset by budesonide-formoterol having lower exacerbation management costs. The ICER of budesonide-formoterol MART was US$7316/QALY from a Chinese society perspective for treating moderate asthma, below the threshold of US$74 024/QALY equivalent to three times gross domestic product (GDP)/capita. Budesonide-formoterol MART was therefore cost-effective compared with ICS-LABA plus SABA reliever.^26^

### Review of methods used

Most studies used clinical data from recent randomised controlled and pragmatic trials.^6 7 28 29^ One study used data from a prospective observational study.^30^ One was a within-randomised controlled trial analysis.^9^ The main differences between studies were the perspectives used (payer vs societal), cost categories included, health outcomes assessed, time horizon and patient population. All studies performed sensitivity or scenario analyses to evaluate the robustness of their results.

## Discussion

This systematic review is the first to present an analysis of the cost-effectiveness of ICS-formoterol AIR/MART for treating mild to moderate asthma globally. Based on findings from six studies, ICS-formoterol AIR/MART was considered cost-effective for the treatment of mild to moderate asthma in adolescents aged 12 years and older and adults. Taken together, these results apply from both a payer and societal perspective. We identified more data for AIR (five studies) than for MART (one study). Identifying fewer data for MART may be due to this study’s focus on mild to moderate asthma treatment (GINA steps 1–3) as opposed to more severe asthma (GINA steps 4–5) where MART is more widely used and studied. We only included results from studies that were assessed as having moderate to high methodological quality as we believed the cost and health outcomes from these studies were credible.

Cost-effectiveness was driven by ICS-formoterol causing fewer exacerbations and adverse events, therefore, reducing the cost of seeking healthcare. This is in line with studies that have shown ICS-formoterol used for the relief of symptoms protects patients from progressing to severe exacerbations, therefore reducing the likelihood of needing emergency department care or hospitalisation, and therefore reducing costs.^31 32^ This is evident whether ICS-formoterol is compared with SABA as required as monotherapy or ICS plus SABA as required.

Globally, poor asthma control is common especially in LMICs. A recent study using data from the Global Asthma Network Phase I found that only 55.4% of adolescents and 61.1% of adults had well-controlled asthma.^33^ In the same study, there was high usage of oral SABA and low usage of ICSs even among those with severe exacerbations. These results match those of another cross-sectional study that found low availability of ICSs (less than 55%) and low affordability when available (less than 35%).^12^ Ensuring people living with asthma have access to affordable, effective ICS-formoterol as AIR or MART would likely contribute to improved asthma control.

Cost-effectiveness analyses complement clinical analyses by identifying opportunity costs that decision makers face, informing resource allocation, and supporting decision makers in selecting medications. Although we found cost-effectiveness data from six countries, due to cost-effectiveness thresholds being dependent on individual country GDPs, these cannot be generalised to all other countries. The difference in medication costs, medical supply chains, and resource availability between countries also contributes to the difficulty of making definite global conclusions.^34^ The six countries represented in this review are in the Americas, European and Western Pacific Regions, while South-East Asian, African and Eastern Mediterranean Regions had no representation. The lack of studies conducted in these regions is concerning as they are made up of mostly LMICs and have prevalence of current asthma symptoms ranging from 4.5% to 15.1% in adolescents.^35^

Among the regions with no data, the African Region has an increasing prevalence of asthma and a disproportionately high number of deaths due to asthma.^36^ Further, this region has a population of more than 1 billion people, with a significant youth population evidenced by over 400 million people in the 15 to 35 age group.^37^ Previously identified barriers to good asthma management include poor patient knowledge about asthma symptoms, underdiagnosis, lack of access to affordable recommended medications, low adherence and stigma.^13^ The region also has a disproportionately low public health expenditure, resulting in constrained health resources and high out-of-pocket health spending.^38^ Using cost-effectiveness data from other settings outside the region may therefore be unsuitable for many African countries and may be less accepted by African decision makers due to a lack of trust in data applicability. Research on the cost-effectiveness of AIR or MART in the region is therefore needed to fill this important evidence gap to inform African policy makers using context-specific and relevant data. A shift to AIR and MART becoming the standard of treatment in these countries could contribute to addressing some of the existing barriers in asthma care.

This was the first systematic review focused on the cost-effectiveness of ICS-formoterol AIR/MART for mild to moderate asthma. A previous systematic review investigated single combination inhalers compared with multiple inhalers for asthma and COPD.^39^ The study did include costs, but interventions included other single inhaler combinations (ICS-SABA, ICS-LABA) and comparators included ICS plus formoterol in separate inhalers. A systematic review from 2018 reviewed the cost-effectiveness of a wider selection of medications for various asthma severities and also supported the cost-effectiveness of ICS-formoterol MART compared with ICS-LABA plus SABA.^40^

### Study strengths and limitations

The strengths of this study include having a systematic search strategy across multiple databases and thorough screening by independent reviewers. We included different perspectives and cost outcomes used in cost-effectiveness analyses to provide comprehensive data. We also assessed risk of bias and only included the findings from studies that were assessed as moderate to high quality. The review focused on the asthma severities that have the largest proportion of patients. The study has several limitations. We found eligible articles from only six countries and therefore could not generalise findings globally due to different cost-effectiveness thresholds between countries. This review only included articles that were available in English. Most studies used Markov models which assume mutually exclusive health states and that transitions between states are independent of time in another state or other previous events, but studies performed sensitivity analyses to assess robustness. Data used for clinical inputs in the lifetime horizon models were from 1-year duration trials and extrapolated to the lifetime horizon.

## Conclusions

For adolescents and adults with mild to moderate asthma, combination ICS-formoterol used as AIR or MART may be cost-effective compared with bronchodilator and ICS inhalers, used separately or in combination, from a payer and society perspective.

## Supplementary material

10.1136/bmjresp-2026-004148 online supplemental file 1

## Data Availability

Data are available in a public, open access repository.
