# Supplementary material for: Cost-effectiveness of anti-inflammatory reliever (AIR) or maintenance and reliever therapy (MART) for mild to moderate asthma: a systematic review
Source: BMJ Open Respir Res. 2026 Oct 8;13(1):e004148. doi: 10.1136/bmjresp-2026-004148 (PMC13656149; doi:10.1136/bmjresp-2026-004148)
Supplement: Supplementary file 1 [file bmjresp-13-1-s001.docx]

**Supplementary Appendix 1**

**Search Terms**

**Population**

Adolescent OR adolescen* OR teenager OR adult OR adult* OR “12 years and older” OR "18 years and older” OR teenage OR teen* OR youth*

AND asthma OR allergic asthma OR asthmatic state OR extrinsic asthma OR intrinsic asthma OR mild asthma OR mild intermittent asthma OR mild persistent asthma OR moderate asthma OR moderate persistent asthma OR asthma* OR wheez*

**Intervention**

Inhaled steroids OR steroids OR systemic steroids OR anti-inflammatory OR anti-inflammatories OR ICS OR corticosteroid OR beclomethasone OR beclomet* OR fluticasone OR “fluticasone mdi” OR “steroid inhaler” OR budesonide OR fluticasone OR fluticasone furoate OR fluticasone propionate OR mometasone OR mometasone furoate

AND formoterol fumarate OR formoterol OR rapid onset long-acting β2-agonist OR rapid long-acting β2-agonist OR fast onset long-acting β2-agonist OR fast acting long-acting β2-agonist OR fast acting beta2-agonist OR long-acting β2-agonist OR β2-agonist OR β-agonist OR combination inhaler OR maintenance OR maintenance and reliever

**Comparator**

Inhaled steroids OR steroids OR systemic steroids OR anti-inflammatory OR anti-inflammatories OR ICS OR corticosteroid OR beclomethasone OR beclomet* OR fluticasone OR “fluticasone mdi” OR “steroid inhaler” OR budesonide OR ciclesonide OR flunisolide OR fluticasone OR fluticasone furoate OR fluticasone propionate OR mometasone OR mometasone furoate

AND albuterol OR levalbuterol OR metaproterenol OR terbutaline OR terbutalin* OR short-acting β2-agonist OR short-acting β-agonist OR SABA OR long-acting β2-agonist OR long-acting β-agonist OR β2-agonist OR β-agonist OR bronchodilator OR LABA OR salmeterol OR salmeterol xinafoate OR vilanterol trifenatate OR indacaterol acetate

OR inhaled steroids OR steroids OR systemic steroids OR anti-inflammatory OR anti-inflammatories OR ICS OR corticosteroid OR beclomethasone OR beclomet* OR fluticasone OR “fluticasone mdi” OR “steroid inhaler” OR budesonide OR ciclesonide OR flunisolide OR fluticasone OR fluticasone furoate OR fluticasone propionate OR mometasone OR mometasone furoate

OR albuterol OR levalbuterol OR metaproterenol OR terbutaline OR terbutalin* OR short-acting β2-agonist OR short-acting β-agonist OR SABA OR β2-agonist OR β-agonist OR bronchodilator

**Outcome**

Cost-effectiveness OR cost utility OR cost benefit OR cost saving OR incremental cost effectiveness ratio OR ICER OR cost analysis OR cost OR economics OR QALY OR asthma control test OR asthma control OR exacerbation OR lung function

**Time**

2013-2023

**Study type**

“Systematic review” OR “comparative observational” OR “observation” OR “model” OR “modelling” OR “Markov cohort model” OR “Markov model” OR “Markov” OR open-controlled, randomised OR meta-analysis OR “metanalysis” OR “Randomised controlled trial” OR “Non-randomised controlled trial” OR “non randomised controlled trial” OR “non randomised” OR “Double-blind, randomised controlled trial” OR Single-blind, “randomised controlled trial” OR randomised OR double blind OR Controlled Clinical Trials, Randomised OR Clinical Trials, Randomised OR Trials, Randomised Clinical OR Meta-Analysis OR Data Pooling OR multicenter study OR crossover OR Overviews, Clinical Trial OR Overview, Clinical Trial OR Clinical Trial Overview* OR Data Pooling*

**Web of Science**

1.	(Adolescent OR adolescen* OR teenagers OR adult OR adult* OR “12 years and older” OR "18 years and older” OR teenage OR teen* OR youth*)

AND (asthma OR “allergic asthma” OR “asthmatic state” OR “extrinsic asthma” OR “intrinsic asthma” OR “mild asthma” OR “mild intermittent asthma” OR “mild persistent asthma” OR “moderate asthma” OR “moderate persistent asthma” OR asthma* OR wheez*)

2.	(“Inhaled steroids” OR steroids OR “systemic steroids” OR “anti-inflammatory” OR “anti-inflammatories” OR ICS OR corticosteroid OR beclomethasone OR beclomet* OR fluticasone OR “fluticasone mdi” OR “steroid inhaler” OR budesonide OR fluticasone OR “fluticasone furoate” OR “fluticasone propionate” OR mometasone OR “mometasone furoate”)

AND (“formoterol fumarate” OR formoterol OR (“rapid onset” AND “long-acting β2-agonist”) OR (rapid AND “long-acting β2-agonist”) OR (“fast onset” AND “long-acting β2-agonist”) OR (“fast acting” AND “long-acting β2-agonist”) OR (“fast acting” AND “beta2-agonist”) OR “long-acting β2-agonist” OR β2-agonist OR β-agonist OR “combination inhaler” OR maintenance OR “maintenance and reliever”)

3.	1 and 2

4.	Cost-effectiveness OR “cost utility” OR “cost benefit” OR “cost saving” OR “incremental cost effectiveness ratio” OR ICER OR “cost analysis” OR cost OR economics OR QALY OR “asthma control test” OR “asthma control” OR exacerbation OR “lung function”

5.	3 and 4

6.	ABS(“Systematic review” OR “comparative observational” OR observation OR model OR modelling OR “Markov cohort model” OR “Markov model” OR Markov OR (open-controlled AND randomised) OR meta-analysis OR metanalysis OR “Randomised controlled trial” OR “Non-randomised controlled trial” OR “non randomised controlled trial” OR “non randomised” OR “Double-blind randomised controlled trial” OR (Single-blind AND “randomised controlled trial”) OR randomised OR “double blind” OR “Controlled Clinical Trials Randomised” OR “Clinical Trials Randomised” OR “Trials Randomised Clinical” OR Meta-Analysis OR “Data Pooling” OR “multicenter study” OR crossover OR (Overviews AND “Clinical Trial”) OR “Overview Clinical Trial” OR “Clinical Trial Overview*” OR “Data Pooling*”)

7.	5 and 6

8.	“inhaled steroids” OR steroids OR “systemic steroids” OR anti-inflammatory OR anti-inflammatories OR ICS OR corticosteroid OR beclomethasone OR beclomet* OR fluticasone OR “fluticasone mdi” OR “steroid inhaler” OR budesonide OR ciclesonide OR flunisolide OR fluticasone OR “fluticasone furoate” OR “fluticasone propionate” OR mometasone OR “mometasone furoate”

9.	albuterol OR levalbuterol OR metaproterenol OR terbutaline OR terbutalin* OR “short-acting β2-agonist” OR “short-acting β-agonist” OR SABA OR β2-agonist OR β-agonist OR bronchodilator

10.	(“Inhaled steroids” OR steroids OR “systemic steroids” OR anti-inflammatory OR anti-inflammatories OR ICS OR corticosteroid OR beclomethasone OR beclomet* OR fluticasone OR “fluticasone mdi” OR “steroid inhaler” OR budesonide OR ciclesonide OR flunisolide OR fluticasone OR “fluticasone furoate” OR “fluticasone propionate” OR mometasone OR “mometasone furoate”)

AND (albuterol OR levalbuterol OR metaproterenol OR terbutaline OR terbutalin* OR “short-acting β2-agonist” OR “short-acting β-agonist” OR SABA OR “long-acting β2-agonist” OR “long-acting β-agonist” OR β2-agonist OR β-agonist OR bronchodilator OR LABA OR salmeterol OR “salmeterol xinafoate” OR “vilanterol trifenatate” OR “indacaterol acetate”)

11.	#8 OR 9 OR 10

12.	#7 and 11

Filtered by year and English

**Cochrane**

1.	(Adolescent OR adolescen* OR teenagers OR adult OR adult* OR “12 years and older” OR “18 years and older” OR teenage OR teen* OR youth*)

AND (asthma OR “allergic asthma” OR “asthmatic state” OR “extrinsic asthma” OR “intrinsic asthma” OR “mild asthma” OR “mild intermittent asthma” OR “mild persistent asthma” OR “moderate asthma” OR “moderate persistent asthma” OR asthma* OR wheez*)

AND (“Inhaled steroids” OR steroids OR “systemic steroids” OR “anti-inflammatory” OR ”anti-inflammatories” OR ICS OR corticosteroid OR beclomethasone OR beclomet* OR fluticasone OR “fluticasone mdi” OR “steroid inhaler” OR budesonide OR fluticasone OR “fluticasone furoate” OR “fluticasone propionate” OR mometasone OR “mometasone furoate”)

AND (“formoterol fumarate” OR formoterol OR (“rapid onset” AND “long acting β2 agonist”) OR (rapid AND “long acting β2 agonist”) OR (“fast onset” AND “long-acting β2 agonist”) OR (“fast acting” AND “long acting β2 agonist”) OR (“fast acting” AND “beta2 agonist”) OR “long acting β2 agonist” OR β2 agonist OR β agonist OR “combination inhaler” OR maintenance OR “maintenance and reliever”)

2.	“inhaled steroids” OR steroids OR “systemic steroids” OR anti-inflammatory OR anti-inflammatories OR ICS OR corticosteroid OR beclomethasone OR beclomet* OR fluticasone OR “fluticasone mdi” OR “steroid inhaler” OR budesonide OR ciclesonide OR flunisolide OR fluticasone OR “fluticasone furoate” OR “fluticasone propionate” OR mometasone OR “mometasone furoate”

OR albuterol OR levalbuterol OR metaproterenol OR terbutaline OR terbutalin* OR “short acting β2 agonist” OR “short acting β agonist” OR SABA OR β2 agonist OR β agonist OR bronchodilator

OR (“Inhaled steroids” OR steroids OR “systemic steroids” OR anti-inflammatory OR anti-inflammatories OR ICS OR corticosteroid OR beclomethasone OR beclomet* OR fluticasone OR “fluticasone mdi” OR “steroid inhaler” OR budesonide OR ciclesonide OR flunisolide OR fluticasone OR “fluticasone furoate” OR “fluticasone propionate” OR mometasone OR “mometasone furoate”)

AND (albuterol OR levalbuterol OR metaproterenol OR terbutaline OR terbutalin* OR “short acting β2 agonist” OR “short acting β agonist” OR SABA OR “long acting β2 agonist” OR “long acting β agonist” OR β2 agonist OR β agonist OR bronchodilator OR LABA OR salmeterol OR “salmeterol xinafoate” OR “vilanterol trifenatate” OR “indacaterol acetate”)

3. Cost-effectiveness OR “cost utility” OR “cost benefit” OR “cost saving” OR “incremental cost effectiveness ratio” OR ICER OR “cost analysis” OR cost OR economics OR QALY OR “asthma control test” OR “asthma control” OR exacerbation OR “lung function”

AND “Systematic review” OR “comparative observational” OR observation OR model OR modelling OR “Markov cohort model” OR “Markov model” OR Markov OR (open-controlled AND randomised) OR meta-analysis OR metanalysis OR “Randomised controlled trial” OR “Non-randomised controlled trial” OR “non randomised controlled trial” OR “non randomised” OR “Double-blind randomised controlled trial” OR (Single-blind AND “randomised controlled trial”) OR randomised OR “double blind” OR “Controlled Clinical Trials Randomised” OR “Clinical Trials Randomised” OR “Trials Randomised Clinical” OR Meta-Analysis OR “Data Pooling” OR “multicenter study” OR crossover OR (Overviews AND “Clinical Trial”) OR “Overview Clinical Trial” OR Clinical Trial Overview* OR Pooling*

4.	#1 and #2 and #3”

Filtered by year and English

Searched in all fields but for the study type searched in abs/key/title

**Scopus**

1.	(Adolescent OR adolescen* OR teenagers OR adult OR adult* OR “12 years and older” OR "18 years and older” OR teenage OR teen* OR youth*)

AND (asthma OR “allergic asthma” OR “asthmatic state” OR “extrinsic asthma” OR “intrinsic asthma” OR “mild asthma” OR “mild intermittent asthma” OR “mild persistent asthma” OR “moderate asthma” OR “moderate persistent asthma” OR asthma* OR wheez*)

2.	(“Inhaled steroids” OR steroids OR “systemic steroids” OR “anti-inflammatory” OR “anti-inflammatories” OR ICS OR corticosteroid OR beclomethasone OR beclomet* OR fluticasone OR “fluticasone mdi” OR “steroid inhaler” OR budesonide OR fluticasone OR “fluticasone furoate” OR “fluticasone propionate” OR mometasone OR “mometasone furoate”)

AND (“formoterol fumarate” OR formoterol OR (“rapid onset” AND “long-acting β2-agonist”) OR (rapid AND “long-acting β2-agonist”) OR (“fast onset” AND “long-acting β2-agonist”) OR (“fast acting” AND “long-acting β2-agonist”) OR (“fast acting” AND “beta2-agonist”) OR “long-acting β2-agonist” OR β2-agonist OR β-agonist OR “combination inhaler” OR maintenance OR “maintenance and reliever”)

3.	1 and 2

4.	Cost-effectiveness OR “cost utility” OR “cost benefit” OR “cost saving” OR “incremental cost effectiveness ratio” OR ICER OR “cost analysis” OR cost OR economics OR QALY OR “asthma control test” OR “asthma control” OR exacerbation OR “lung function”

5.	3 and 4

6.	“Systematic review” OR “comparative observational” OR observation OR model OR modelling OR “Markov cohort model” OR “Markov model” OR Markov OR (open-controlled AND randomised) OR meta-analysis OR metanalysis OR “Randomised controlled trial” OR “Non-randomised controlled trial” OR “non randomised controlled trial” OR “non randomised” OR “Double-blind randomised controlled trial” OR (Single-blind AND “randomised controlled trial”) OR randomised OR “double blind” OR “Controlled Clinical Trials Randomised” OR “Clinical Trials Randomised” OR “Trials Randomised Clinical” OR Meta-Analysis OR “Data Pooling” OR “multicenter study” OR crossover OR (Overviews AND “Clinical Trial”) OR “Overview Clinical Trial” OR “Clinical Trial Overview*” OR “Data Pooling*”

7.	5 and 6

8.	“inhaled steroids” OR steroids OR “systemic steroids” OR anti-inflammatory OR anti-inflammatories OR ICS OR corticosteroid OR beclomethasone OR beclomet* OR fluticasone OR “fluticasone mdi” OR “steroid inhaler” OR budesonide OR ciclesonide OR flunisolide OR fluticasone OR “fluticasone furoate” OR “fluticasone propionate” OR mometasone OR “mometasone furoate”

9.	albuterol OR levalbuterol OR metaproterenol OR terbutaline OR terbutalin* OR “short-acting β2-agonist” OR “short-acting β-agonist” OR SABA OR β2-agonist OR β-agonist OR bronchodilator

10.	(“Inhaled steroids” OR steroids OR “systemic steroids” OR anti-inflammatory OR anti-inflammatories OR ICS OR corticosteroid OR beclomethasone OR beclomet* OR fluticasone OR “fluticasone mdi” OR “steroid inhaler” OR budesonide OR ciclesonide OR flunisolide OR fluticasone OR “fluticasone furoate” OR “fluticasone propionate” OR mometasone OR “mometasone furoate”)

AND (albuterol OR levalbuterol OR metaproterenol OR terbutaline OR terbutalin* OR “short-acting β2-agonist” OR “short-acting β-agonist” OR SABA OR “long-acting β2-agonist” OR “long-acting β-agonist” OR β2-agonist OR β-agonist OR bronchodilator OR LABA OR salmeterol OR “salmeterol xinafoate” OR “vilanterol trifenatate” OR “indacaterol acetate”)

11.	#8 OR 9 OR 10

12.	#7 and 11

Filtered by year and English

Refined by TITLE-ABS-KEY

**Pubmed**

1.	(Adolescent OR adolescen* OR teenagers OR adult OR adult* OR “12 years and older” OR “18 years and older” OR teenage OR teen* OR youth*)

AND (asthma OR “allergic asthma” OR “asthmatic state” OR “extrinsic asthma” OR “intrinsic asthma” OR “mild asthma” OR “mild intermittent asthma” OR “mild persistent asthma” OR “moderate asthma” OR “moderate persistent asthma” OR asthma* OR wheez*)

2.	(“Inhaled steroids” OR steroids OR “systemic steroids” OR “anti-inflammatory” OR “anti-inflammatories” OR ICS OR corticosteroid OR beclomethasone OR beclomet* OR fluticasone OR “fluticasone mdi” OR “steroid inhaler” OR budesonide OR fluticasone OR “fluticasone furoate” OR “fluticasone propionate” OR mometasone OR “mometasone furoate”)

AND (“formoterol fumarate” OR formoterol OR (“rapid onset” AND “long-acting β2-agonist”) OR (rapid AND “long-acting β2-agonist”) OR (“fast onset” AND “long-acting β2-agonist”) OR (“fast acting” AND “long-acting β2-agonist”) OR (“fast acting” AND “beta2-agonist”) OR “long-acting β2-agonist” OR β2-agonist OR β-agonist OR “combination inhaler” OR maintenance OR “maintenance and reliever”)

3.	1 and 2

4.	Cost-effectiveness OR “cost utility” OR “cost benefit” OR “cost saving” OR “incremental cost effectiveness ratio” OR ICER OR “cost analysis” OR cost OR economics OR QALY OR “asthma control test” OR “asthma control” OR exacerbation OR “lung function”

5.	3 and 4

6.	“Systematic review” OR “comparative observational” OR observation OR model OR modelling OR “Markov cohort model” OR “Markov model” OR Markov OR (open-controlled AND randomised) OR meta-analysis OR metanalysis OR “Randomised controlled trial” OR “Non-randomised controlled trial” OR “non randomised controlled trial” OR “non randomised” OR “Double-blind randomised controlled trial” OR (Single-blind AND “randomised controlled trial”) OR randomised OR “double blind” OR “Controlled Clinical Trials Randomised” OR “Clinical Trials Randomised” OR “Trials Randomised Clinical” OR Meta-Analysis OR “Data Pooling” OR “multicenter study” OR crossover OR (Overviews AND “Clinical Trial”) OR “Overview Clinical Trial” OR “Clinical Trial Overview*” OR “Data Pooling*”

7.	5 and 6

8.	“inhaled steroids” OR steroids OR “systemic steroids” OR anti-inflammatory OR anti-inflammatories OR ICS OR corticosteroid OR beclomethasone OR beclomet* OR fluticasone OR “fluticasone mdi” OR “steroid inhaler” OR budesonide OR ciclesonide OR flunisolide OR fluticasone OR “fluticasone furoate” OR “fluticasone propionate” OR mometasone OR “mometasone furoate”

9.	albuterol OR levalbuterol OR metaproterenol OR terbutaline OR terbutalin* OR “short-acting β2-agonist” OR “short-acting β-agonist” OR SABA OR β2-agonist OR β-agonist OR bronchodilator

10.	(“Inhaled steroids” OR steroids OR “systemic steroids” OR anti-inflammatory OR anti-inflammatories OR ICS OR corticosteroid OR beclomethasone OR beclomet* OR fluticasone OR “fluticasone mdi” OR “steroid inhaler” OR budesonide OR ciclesonide OR flunisolide OR fluticasone OR “fluticasone furoate” OR “fluticasone propionate” OR mometasone OR “mometasone furoate”)

AND (albuterol OR levalbuterol OR metaproterenol OR terbutaline OR terbutalin* OR “short-acting β2-agonist” OR “short-acting β-agonist” OR SABA OR “long-acting β2-agonist” OR “long-acting β-agonist” OR β2-agonist OR β-agonist OR bronchodilator OR LABA OR salmeterol OR “salmeterol xinafoate” OR “vilanterol trifenatate” OR “indacaterol acetate”)

11.	#8 OR 9 OR 10

12.	#7 and 11

Filtered by year and English

**Google Scholar**

Advanced search

Keywords: Adolescent|teen|adult|youth|asthma|wheez*|Inhaled|steroid|anti-inflammatory|ICS formoterol|rapid|“fast onset” “long-acting β2-agonist”|β2-agonist|β-agonist|maintenance Cost-effectiveness|“cost utility”|cost|“cost saving”|ICER|“cost analysis”

Years: 2013-2026

**Supplementary Appendix 2**

Risk assessment summary review

| **Study** | **I1** | **I2** | **I3** | **I4** | **I5** | **I6** | **I7** | **I8** | **I9** | **I10** | **I11** | **Overall** |
| --- | --- | --- | --- | --- | --- | --- | --- | --- | --- | --- | --- | --- |
| Buendía [20] | 1 | 1 | 1 | 1 | 0 | 0 | 0 | 1 | 1 | 0 | 0 | 55% |
| Buendía [21] | 1 | 1 | 1 | 0 | 0 | 0 | 0 | 1 | 1 | 0 | 0 | 45% |
| FitzGerald [22] | 1 | 1 | 1 | 1 | 1 | 1 | 1 | 1 | 1 | 1 | 0 | 91% |
| Sadatsafavi [23] | 1 | 1 | 1 | 1 | 1 | 1 | 1 | 1 | 1 | 1 | 1 | 100% |
| Raja Gopal [24] | 1 | 1 | 1 | 1 | 1 | 0 | 1 | 0 | 1 | 1 | 1 | 82% |
| Zhou [26] | 1 | 1 | 1 | 1 | 1 | 0 | 0 | 1 | 1 | 1 | 1 | 82% |
| Rodríguez-Martínez [25] | 1 | 1 | 1 | 1 | 1 | 0 | 0 | 1 | 1 | 1 | 0 | 73% |
| Leung [27] | 1 | 1 | 1 | 1 | 1 | 1 | 1 | 1 | 0 | 1 | 0 | 82% |
